# Equity in health care in Namibia: developing a needs-based resource allocation formula using principal components analysis

**DOI:** 10.1186/1475-9276-6-3

**Published:** 2007-03-29

**Authors:** Eyob Zere, Custodia Mandlhate, Thomas Mbeeli, Kalumbi Shangula, Kauto Mutirua, William Kapenambili

**Affiliations:** 1 World Health Organization, P.O. Box 30309, Lilongwe, Malawi; 2 World Health Organization, P.O. Box 3444, Windhoek, Namibia; 3 Ministry of Health and Social Services, Private Bag 13198, Windhoek, Namibia

## Abstract

**Background:**

The pace of redressing inequities in the distribution of scarce health care resources in Namibia has been slow. This is due primarily to adherence to the historical incrementalist type of budgeting that has been used to allocate resources. Those regions with high levels of deprivation and relatively greater need for health care resources have been getting less than their fair share. To rectify this situation, which was inherited from the apartheid system, there is a need to develop a needs-based resource allocation mechanism.

**Methods:**

Principal components analysis was employed to compute asset indices from asset based and health-related variables, using data from the Namibia demographic and health survey of 2000. The asset indices then formed the basis of proposals for regional weights for establishing a needs-based resource allocation formula.

**Results:**

Comparing the current allocations of public sector health car resources with estimates using a needs based formula showed that regions with higher levels of need currently receive fewer resources than do regions with lower need.

**Conclusion:**

To address the prevailing inequities in resource allocation, the Ministry of Health and Social Services should abandon the historical incrementalist method of budgeting/resource allocation and adopt a more appropriate allocation mechanism that incorporates measures of need for health care.

## Background

In sub-Saharan Africa, health sector reforms have been under way since the 1980s. One of the parameters used to assess the effectiveness of reforms in achieving their stated objectives has been their effect on equity. Equity was enshrined in the World Health Organization's Alma Ata declaration as one of the pillars of the Primary Health Care (PHC) strategy [1]. Equity in access to health care and other social amenities is also one of the tenets of the Universal Declaration of Human Rights. Hence, albeit with varying degrees of explicitness and clarity, the concept and principles of equity feature in the health policies of most countries.

As part of its PHC strategy, the Namibian Ministry of Health and Social Services (MOHSS) considers equity as one of its guiding principles in allocating health care resources. This is then reflected in their desire to improve the health outcomes of the poor and disadvantaged.

In its health policy framework, the MOHSS makes the following statement on equity [2]:

"*All Namibians shall have equal access to basic health care & social services provided by the Ministry. Particular emphasis shall be paid to resource distribution patterns in Namibia to identify and accelerate the correction of disparities"*.

Furthermore, the country's Poverty Reduction Strategy (PRS) commits the government to reduce inter-regional disparities in health expenditure per capita through an appropriate resource allocation formula [3]. However, despite the government's initiatives and equity-orientated health and social policies, there has not been a significant reallocation of resources in favour of the poor regions. The pace of change in decreasing inequities in resource allocation inherited from the apartheid era has not been as fast as was expected. This is explained at least in part by the fact that resources are allocated on the basis of an historical incrementalist type of budgeting. Those regions which are historically disadvantaged in having a higher disease burden, continue to receive an inequitably small share of the health care budget.

Namibia is one of those countries with high levels of inequality in both income and access to resources, including health care. The country's gini index, which is 70.7, is the highest recorded measure of income inequality in the world [4]. The glaring inequalities in income, in access to resources more generally and in health outcomes that are observed in the country are partly attributable to the exclusionary policies of the apartheid regime that prevailed before the country gained its independence in 1990. Although the existence of high levels of inequalities is not unexpected given the country's history, there is a need to generate a solid evidence base to guide future policies for resource allocation to attempt to reduce these inequalities. Health care resources need to be redistributed to those regions with greater levels of need,. This entails a move away from the traditional historical incrementalist basis for allocating resources.

In turn to monitor the impact of policies that are aimed at improving the status of the disadvantaged and measure such changes involves first identifying the poor and vulnerable and second developing appropriate criteria for allocating resources. 'Appropriate' here means based on some assessment of need. Well-targeted health resources can contribute to the improvement of the health status of the worse off in the population, to the achievement of the Millennium Development Goals (MDGs) and to the targets of the country's own initiatives on equity – Vision 2030 and National Development Plans.

The study reported here has been conducted to provide this information to shed light on how an improved mechanism for health care resource allocation in the public health sector in Namibia might result from the development of an equitable funding formula. The more specific objective of the study is to devise regional weightings for the development of such a needs-based resource allocation formula.

Undertaking such a study is significant and relevant for Namibia because it is in line with government policies aimed at reducing inequities in health and health care. The information generated will contribute to policy changes that will assist in bridging the present inequities in the allocation of health care resources.

### Brief country profile

Namibia is a country in south-western Africa covering an area of 824,116 square kilometres. According to the 2001 Census, the country's population was a little more than 1.8 million with a growth rate of 2.6 per cent per annum [5]. The country's health and development indicators are depicted in Table 1. As can be seen, most of the health and development indicators are considerably better than those observed in the rest of sub-Saharan Africa. However, it is worthwhile assessing changes in the indicators to appreciate the magnitude of the health and development challenges that the country faces. The life expectancy at birth of males and females in 1991 was 59 and 63 years respectively. The table shows a decrease from then in the order of 11 to 13 years respectively. This decline is largely attributable to the HIV/AIDS epidemic. Furthermore, the Human Development Index (HDI) which is an indicator of society's welfare as measured by a composite index that includes GDP per capita, longevity and education has declined substantially to 0.627 from its 1995 level of 0.693.

**Table 1:** Namibia – health and development indicators

Characteristic	Value
Life expectancy at birth (male/female) (years)	48/50
Infant mortality rate (per 1000 live births)	38
Under-five mortality rate (per 1000 live births)	62
Total fertility rate	4.2
Maternal mortality ratio (per 100,000 live births)	271
One year olds fully immunized against measles, 2003 (%)	70
Stunting in under-five children (%)	24.0
Gross national income per capita, 2003 (US$)	2,120
GDP per capita annual growth rate 1990–2003 (%)	0.9
Population living below US$1 a day, 1990–2003 (%)	34.9
Human development index, 2003	0.627
Adult literacy rate (15 years and above), 2003 (%)	85
HIV prevalence rate (%)	19.8
Prevalence of tuberculosis (per 100,000)	635
Malaria mortality rate (per 100,000)	39
Per capita total expenditure on health (PPP US$)	331
Health expenditure as % of GDP	6.7
Physician per 100,000 population	30

Sources: [5-11]

The figures in the above table are population averages and therefore mask the wide differences in the indicators in the different population groups. Disaggregating some of the above figures by socio-economic status reveals the dual nature of the Namibian society, where there are characteristics of both developed and developing countries. While the HDI for the European language speakers in Namibia is above the minimum point for what is classed as 'high human development', for some language groups such as the San-speaking people, the HDI is even lower than the average for sub-Saharan Africa [12]. The share of income of the richest 10 per cent of the population is 129 times that of the poorest decile [4]. While the prevalence of stunting in the lowest wealth quintile is about 26.7 per cent, it is only 15.3 per cent for the richest income quintile. Thus, even though the population averages of the indicators are favourable compared to those of other countries in sub-Saharan Africa, these can be misleading. They conceal the fact that many people are systematically excluded from access to the nation's resources.

In general communicable diseases account for the greatest share of the disease burden. However, facility-based statistics also indicate that non-communicable diseases are on the increase, thus compounding the problems for the country's health system. To address the multitude of challenges facing Namibia, the government has put in place various multi-sectoral initiatives. These include the Vision 2030, 5-year National Development Plans (NDPs), a poverty reduction strategy and action programme and medium term plans for the control of HIV/AIDS. Furthermore, there has been a gradual move towards performance-based budgeting and medium-term expenditure frameworks (MTEFs) in order to improve the efficiency of the overall economy.

### Equity in health care: steps in the development of a needs-based resource allocation formula

The concept of equity in health care has been widely debated over the years. Although equity may be defined in many ways, all of its definitions revolve around a common point: the fair distribution of something (such as health services) among different individuals and groups in society. Resource allocation refers to the process by which available resources are distributed among competing needs. It is a means of achieving the ministry's objective of making access to basic health services more equal and ultimately improving health status. Some of the approaches used in resource allocation include political negotiation, incremental budgeting and allocation according to health care needs. The focus of this study is on developing a needs-base resource allocation formula. It is therefore appropriate to spell out the steps involved in its development. These are [13]:

#### (i) Developing a clear operational definition of equity

Central to such a definition is the notion of fairness or justice; however, countries may define equity in different ways. Whatever definition is adopted then influences the steps involved in achieving an equitable allocation of resources. The MOHSS definition discussed above is limited in that it does not take into account differential health needs nor does it provide a clear definition of *access*. For the purposes of this study equity in health care is defined as equal access to a basic package of services for equal need, where:

- *Need *refers to both the "capacity to benefit" and the "severity of illness"; and

- *Access *refers to the barriers, mainly financial and geographical, faced by potential users.

The focus of the above definition is on horizontal equity: equal treatment for equal needs. This implies that the health care system must deal with two individuals with the same complaint in the same way. Given the historical imbalances in Namibia, resources are currently concentrated in the regions with relatively less need. Therefore to achieve a more equitable allocation entails the distribution of more resources to the regions that were historically disadvantaged. There are two ways to do this. One is simply to allocate pro rata with need as the size of the problem which is a form of horizontal equity but which will nonetheless in time reduce inequities. Alternatively one can adopt a vertical equity approach based on the notion of capacity to benefit but where such capacity to benefit is weighted more highly in disadvantaged areas. The latter will clearly reduce inequities faster than the former. While ideally, given the very great differences in health status that exist across different groups in the population, it is vertical equity that should be the basis of equity in healthcare in Namibia, we have adopted a more pragmatic, more politically acceptable horizontal equity approach. To move from where Namibia currently is to vertical equity is politically just too big a step in one go. Nonetheless the authors recommend that in time a formula embracing vertical equity will need to be adopted if real in roads are to be made in equity in Namibian health care.

### (ii) Developing a needs-based formula

The size of the population in a geographic area is the primary indicator of need for health services. Population size can then be weighted by a range of other indicators of relative need such as the demographic composition of the population; amounts of sickness; the level of deprivation (in so far as this influences the level of ill-health in an area); the communities' ability to pay for health care costs; and their level of dependence on public sector health services [14].

#### (iii) Differing costs of service provision

There may be variation in the cost of delivering similar services in different geographic areas of a country. This is likely to be related to input prices (e.g. labour costs) and rough terrain/remoteness, which may inflate transportation costs and increase staff remuneration (relief/hardship allowance). Furthermore, this may also reduce input productivity and consequently increase costs. Therefore, it is important to accommodate such cost differentials in allocating resources.

#### (iv) Other funding sources

The attainment of equal access to basic health services entails taking into account other funding sources such as payments from private households, insurance and development partners. For example in South Africa, members of medical aid schemes are excluded from the base population used for resource allocation purposes [13]. However, this requires that accurate expenditure data are available. A number of countries including Namibia are currently trying to address this issue through a National Health Accounts (NHA) exercise.

In developing countries in general there remain problems in developing needs-based resource allocation formulae. These include:

1. Lack of reliable and timely data.

2. The tendency to create perverse incentives, e.g. that exaggerate the size of the population or to seek to influence other factors that make up any allocation formula.

3. The exclusion of certain services from the formula can become problematic. For example some essential services may be considered national services, hereas others may be considered as regional or district services. There is often no clear agreement about such definitions.

4. Defining what is contained in the basic package of services may be difficult. Additionally it may be the case that the package does not address the needs of the poor which is then contrary to the principle of equitable resource allocation.

### Study methodology

#### Data Sources

Data used for the study were obtained from the Namibia Demographic and Health Survey (DHS) 2000. These were taken as proxies for household income/consumption data as these latter do not in fact exist.

#### Data analysis

The indicator of need was based on the computation of asset indices for each Household. These take into account a set of asset-based and health-related variables.

The most accurate poverty indices would be computed from household consumption data. The absence of such information in the DHS however precludes its use. Therefore there is a need to rely on some other data. Studies have shown that there is a close relationship between asset indices and consumption expenditure. (See for example Filmer and Pritchett [15], in their study on the education sector in India.) Hence in the absence of reliable household income and expenditure data, the use of asset indices becomes important.

These included:

- Whether a household has electricity, radio, television, refrigerator, any bicycles, any motorcycles, a car or a telephone (each coded as 1 = Yes and 0 = No);

- The main household source of drinking water (seven categories);

- The main type of toilet facility used by the household (six categories); and

- The main type of floor in the household (five categories).

The method of principal components analysis (PCA) was used to determine the weights of asset indices based on the variables listed above. PCA is helpful when we have obtained data on many variables and wish to develop a smaller number of (possibly) artificial variables (principal components) that will explain most of the variance in the observed variables. In PCA it is assumed that there is some redundancy in the variables collected, implying that some of the variables are correlated with one another and are possibly measuring the same thing. The first principal component is the linear combination of variables with the largest amount of information common to all of the variables. The greatest weakness of the PCA is the lack of theory to motivate either the choice of variables or the appropriateness of the weights generated.

The result obtained from the first principal component is usually used to develop the asset index based on the following formula:

Aj=f1×(aj1−a1s1)+...+fn×(ajn−ansn)
 MathType@MTEF@5@5@+=feaafiart1ev1aaatCvAUfKttLearuWrP9MDH5MBPbIqV92AaeXatLxBI9gBaebbnrfifHhDYfgasaacH8akY=wiFfYdH8Gipec8Eeeu0xXdbba9frFj0=OqFfea0dXdd9vqai=hGuQ8kuc9pgc9s8qqaq=dirpe0xb9q8qiLsFr0=vr0=vr0dc8meaabaqaciaacaGaaeqabaqabeGadaaakeaacqWGbbqqdaWgaaWcbaGaemOAaOgabeaakiabg2da9iabdAgaMnaaBaaaleaacqaIXaqmaeqaaOGaey41aq7aaeWaaeaadaWcaaqaaiabdggaHnaaBaaaleaacqWGQbGAcqaIXaqmaeqaaOGaeyOeI0Iaemyyae2aaSbaaSqaaiabigdaXaqabaaakeaacqWGZbWCdaWgaaWcbaGaeGymaedabeaaaaaakiaawIcacaGLPaaacqGHRaWkcqGGUaGlcqGGUaGlcqGGUaGlcqGHRaWkcqWGMbGzdaWgaaWcbaGaemOBa4gabeaakiabgEna0oaabmaabaWaaSaaaeaacqWGHbqydaWgaaWcbaGaemOAaOMaemOBa4gabeaakiabgkHiTiabdggaHnaaBaaaleaacqWGUbGBaeqaaaGcbaGaem4Cam3aaSbaaSqaaiabd6gaUbqabaaaaaGccaGLOaGaayzkaaaaaa@566C@

In the above formula:

*A*_*j *_is the asset index of the *j*^*th *^household

*f*_1 _is the scoring factor for the first asset as determined by the procedure;

*a*_*j*1 _is the *j*^*th *^household's value for the first asset; and

*a*_1 _and *s*_1 _are the mean and standard deviation of the first asset variable over all households.

Data were analysed using STATA/SE 8.2 and Microsoft excel.

## Results

The asset and health-related variables used in the PCA model showed a pattern that is consistent with what would have been expected. The assets that are likely to be owned by the better-off household have positive values, which increase the household's asset index. On the other hand, those that characterize poor households (e.g. a pit latrine and an open well) have negative values, which result in a decreased asset index. The scoring factors from the PCA model are set out in Table 2.

**Table 2:** Scoring factors

Variable	Score
Electricity	0.3340
Radio	0.1332
Refrigerator	0.3274
Television	0.3116
Bicycle	0.0454
Motorbike	0.0588
Car	0.1627
Telephone	0.2604
Piped water	0.0000
Open well	-0.3904
Surface water	-0.2856
Borehole	-0.2637
Rain water	-0.3455
Tanker	-0.0314
Other water	-0.1499
Flush toilet	0.0000
Pit latrine	-0.1802
VIP* latrine	-0.1206
Bucket latrine	-0.0626
No toilet	-0.6575
Other toilet	-0.0308
Natural floor	0.0000
Wood floor	0.0670
Ceramic floor	0.4376
Cement floor	0.4376
Carpet	0.3421

* Ventillated Improved Pit latrine

These scoring factors were fed into Equation 1 to derive the asset indices. In other words, the products of the scoring factors and the standardized value of the asset and health-related variables give the asset index. For this procedure, the indices for the 13 regions were computed. These are presented in Table 3. It should be noted that it was not possible to compute indices for the 34 health districts, as data do not allow this. The index values depicted in Table 3 have negative values for regions that are relatively less deprived and positive for those that are relatively more deprived. It can be seen that regions such as Ohangwena, Omusati and Caprivi have the highest positive values indicating the presence of relatively higher levels of deprivation.

**Table 3:** Asset indices by region

Region	Index
Caprivi	0.1328
Erongo	0.0138
Hardap	-0.0417
Karas	-0.0093
Khomas	0.1084
Kunene	-0.0032
Ohangwena	0.2481
Kavango	0.0465
Omaheke	0.0039
Omusati	0.1730
Oshana	0.0609
Oshikoto	0.0584
Otjozondjupa	-0.0225

In deriving a resource allocation formula that includes asset indices, there is a need to normalize the indices in Table 3[14]. This means giving the least deprived region a value of 1 and expressing all other regions in relation to that value. To this end, we added 1.0417 to all the asset indices of the regions – a figure that makes the value of the least deprived region (Hardap) equal to 1. The resultant values were used as weights to adjust the population of each region for resource allocation purposes. These values are presented in Table 4.

**Table 4:** Normalized asset indices and weighted population

Region	Normalized asset index	Population 2001	Population weighted by asset index	Share of unweighted population (%)	Share of weighted population (%)
Caprivi	1.1747	79,826	93,771	4.4	4.5
Erongo	1.0489	107,663	112,923	5.9	5.5
Hardap	1	68,249	68,249	3.7	3.3
Karas	1.0344	69,329	71,717	3.8	3.5
Khomas*	1.1387	250,262	284,978	13.7	13.8
Kunene	1.0377	68,735	71,330	3.8	3.5
Ohangwena	1.2889	228,384	294,373	12.5	14.3
Kavango*	1.0901	202,694	220,968	11.1	10.7
Omaheke	1.0530	68,039	71,648	3.7	3.5
Omusati	1.2162	228,842	278,324	12.5	13.5
Oshana*	1.1087	161,916	179,513	8.8	8.7
Oshikoto	1.0996	161,007	177,051	8.8	8.6
Otjozondjupa	1.0199	135,384	138,073	7.4	6.7

* Based on personal communication with hospital management, the following assumptions on population from the immediate catchment area were used in allocating the budget for referral hospitals in these regions: Khomas (Windhoek Central hospital = 15%, Katatura hospital = 50%); Kavango (Rundu hospital = 80%); and Oshana (Oshakati hospital = 50%)

As can be observed from Table 4, the regional share of weighted population increases for those regions that are relatively more deprived. For example, the unweighted share of population for Ohangwena is 12.5%. However, after weighting with the normalized asset index, the share increased to 14.3%. In other words, if we allocate resources on the basis of weighted population, the allocation share of Ohangwena region will increase by about 2 percentage points. This is an adjustment to the region's increased needs as indicated by the asset indices.

**Figure 1:**
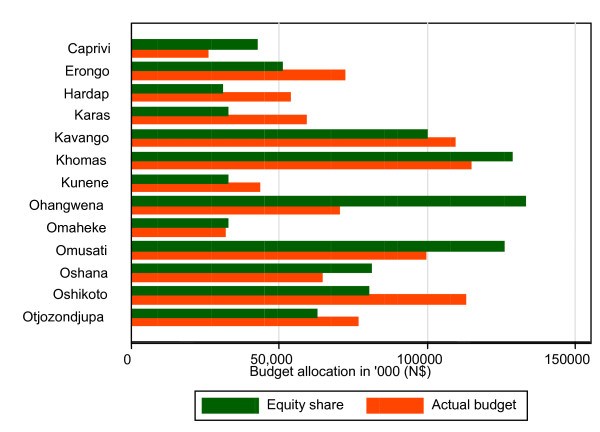
**Equity share vs. actual budget for 2000/2001 financial year**. NB: The budgets do not include central administrative costs, as the central Ministry of Health and Social Services is a supra-regional entity.

Thus, in allocating resources based on the asset index, it is important that we focus on weighted population in order to take account of the differential needs for health care. To illustrate this, Figure 1 presents the actual allocation of the 2000/2001 financial year's budget of the Ministry of Health and Social Services and contrasts them with the equity target share of the budget.

As can be seen from Figure 1, there are major inequities in the distribution of public sector healthcare resources between regions. For example, Ohangwena, which is relatively speaking the most deprived region, receives a budget that is about 89% less than it would get with its equitable share. On the other hand, Hardap, which is the least deprived region, receives a budget that is about 73% more than its equitable share.

## Discussion

This study has attempted to shed light on issues of health resource allocation in Namibia, with the aim of generating the evidence required to move away from historical incrementalism towards a needs-based allocation. Based on data from DHS 2000, PCA was used to develop asset indices as proxies for the regional population's need for health care. Indices were computed using asset-based and health-related variables. These largely reflect levels of material deprivation. However, given the well-established relationship between income poverty and indicators of health status, the asset indices may be considered as proxies for health care needs.

Due to lack of data on micro-geographic areas (e.g. health districts, constituencies, etc.), the study has focussed on developing criteria to allocate resources among the thirteen regions. The analysis at regional level has its limitations in that the population is unlikely to be homogeneous in its characteristics across a whole region, implying that there are going to be deprived areas within relatively well-off regions. Therefore it is desirable to establish district-level databases to promote the equitable allocation of resources within regions.

The results of the PCA model have identified those regions that are regarded as relatively poor in line with the findings of other studies such as the Namibia Human Development Report, 2000/2001 [12]. The model identified Caprivi, Ohangwena and Omusati regions as being the most deprived. The same regions emerge as being worst-off in terms of their human development indices. There is a similar agreement between the two in identifying the least deprived regions. The human development index captures material and social welfare, as it is composed of per capita income, education (literacy rate and school enrolment) and longevity.

The fact that the results of the PCA model agree with the human development indices adds to the credibility of the model as a measure of not only material but also social deprivation. The study reveals inequities at present in the distribution of health care resources between regions. The regions with more need for health care currently get a lower share of the public sector resources, while those with relatively less need are allocated a greater share of resources. This is in line with the inverse care law. Unless the current system of resource allocation is changed to take account of differential regional needs, inequities that were inherited from the past will be perpetuated. In Namibia, the current allocation of resources falls short of equal expenditure per capita. Therefore moving towards needs-based allocation will take some time. In the interim, it is recommended that measures be taken towards achieving equal expenditure per capita. This should be done through a levelling-up approach, to avoid any negative consequences on the health system, in terms of either health or politically.

## Competing interests

The author(s) declare that they have no competing interests.

## Authors' contributions

EZ designed the study, performed the analysis and drafted the report; TM participated in the collection of data, analysis and write-up of the report; KS, KM and CM participated in the write-up of the report; WK participated in data collection and report write-up; All authors read and approved the final manuscript.
